# Supplementary material for: Evolution of urban scaling: Evidence from Brazil
Source: PLoS One. 2018 Oct 4;13(10):e0204574. doi: 10.1371/journal.pone.0204574 (PMC6171854; doi:10.1371/journal.pone.0204574)
Supplement: S1 Table — Description of the studied variables containing units, expected scaling regime [10] and source. (PDF) [file pone.0204574.s001.pdf]

Supporting information

S1 Table Studied variables. Description of the studied variables containing units, expected scaling regime [10] and source.

Variable	Unit	Scaling Regime	Source
Population	number	base	IBGE/census
Surface of Administrative Area	Km2	infrastructure	IBGE/census
Gross Domestic Product	R$	social output	IPEA
Lenght of Street Network	Km	infrastructure	OSM
Number of Health Facilities	number	infrastructure	IBGE/cities
numberOfHospitalBeds	number	infrastructure	IBGE/cities
numberOfDaycareFacilities	number	infrastructure	IBGE/cities
numberOfPrimarySchools	number	infrastructure	IBGE/cities
numberOfSecondarySchools	number	infrastructure	IBGE/cities
numberOfNonGovernmentalOrganizations	number	social output	IBGE/cities
numberOfCommercialEnterprises	number	infrastructure	IBGE/cities
numberOfCommercialEnterprisesFacility	number	infrastructure	IBGE/cities
numberOfDeathsByTrafficAccident	number	social output	DATASUS
numberOfHomicides	number	social output	DATASUS
numberOfSuicides	number	social output	DATASUS
currentExpenditure	R$	infrastructure	IPEA
subsidyExpenditure	R$	infrastructure	IPEA
capitalExpenditure	R$	infrastructure	IPEA
budgetedExpenditure	R$	infrastructure	IPEA
expenditureByFunction	R$	infrastructure	IPEA
Transfer Expenditure	R$	social output	IPEA
budgetedRevenue	R$	social output	IPEA
currentRevenue	R$	social output	IPEA
taxRevenue	R$	social output	IPEA
capitalRevenue	R$	social output	IPEA
taxRevenueTaxes/taxes	R$	social output	IPEA
taxRevenueUrbanLandTax	R$	social output	IPEA
taxRevenueServiceTax	R$	social output	IPEA
taxRevenueTax- Rates	R$	social output	IPEA
numberOfRegisteredInhabitants	number	social output	IBGE/census
numberOfLiterateInhabitants	number	social output	IBGE/census
numberOfHousesWithBathroom	number	base	IBGE/census
numberOfHousesConnectedToSewageSystem	number	base	IBGE/census
numberOfInhabitantsWithMunicipalWaterSupply	number	base	IBGE/census
numberOfInhabitantsServedByWasteCollection	number	base	IBGE/census
numberOfInhabitantsWithAccessToElectricity	number	base	IBGE/census
numberOfInhabitantsWithExclusiveBathroom	number	base	IBGE/census
numberOfInhabitantsWithElectricityMeasurement	number	base	IBGE/census
numberOfInhabitantsWithWasteIllegalDumping	number	base	IBGE/census
numberOfInhabitantsWithWasteIllegalBurning	number	base	IBGE/census
Water and Sewage systems/Total revenue	R$	infrastructure	SNIS
Water and Sewage systems/Total expenditures	R$	infrastructure	SNIS
Water and Sewage systems/Staff expenditures	R$	infrastructure	SNIS
Population with water supply	number	infrastructure	SNIS
Water suply network/links	number	infrastructure	SNIS
Water suply network/length	Km	infrastructure	SNIS
Water suply/electricity consumption	KhH/year	infrastructure	SNIS
Water suply/investments	R$/year	infrastructure	SNIS
Water suply/impacted consumers shutdowns	number	infrastructure	SNIS
Population with sewage collection	number	base	SNIS
Sewage collection network/links	number	infrastructure	SNIS
Sewage collection network/length	Km	infrastructure	SNIS
Sewage collection/collected volume	km3/year	base	SNIS
Sewage collection/wastewater analyzed samples	number	infrastructure	SNIS
Waste collection/attended population	number	base	SNIS
Waste collection/assoiated garbage collectors	number	infrastructure	SNIS
Waste collection/waste collected	Tons/year	base	SNIS
Waste collection/waste trucks	number	infrastructure	SNIS
Waste collection/total expenditures		infrastructure	SNIS
Waste collection/total workers	number	infrastructure	SNIS
Waste collection/swept sidewalks	Km	social output	SNIS
