# Supplementary material for: Evolution of urban scaling: Evidence from Brazil
Source: PLoS One. 2018 Oct 4;13(10):e0204574. doi: 10.1371/journal.pone.0204574 (PMC6171854; doi:10.1371/journal.pone.0204574)

S1 Fig Scaling exponent β as a function of minimum density cut-off for all the variables. Each line represents the scaling exponent (y-axis) from OLS regressions of the log-transformed data of each variable as a function of the minimum density cut-off (x-axis).

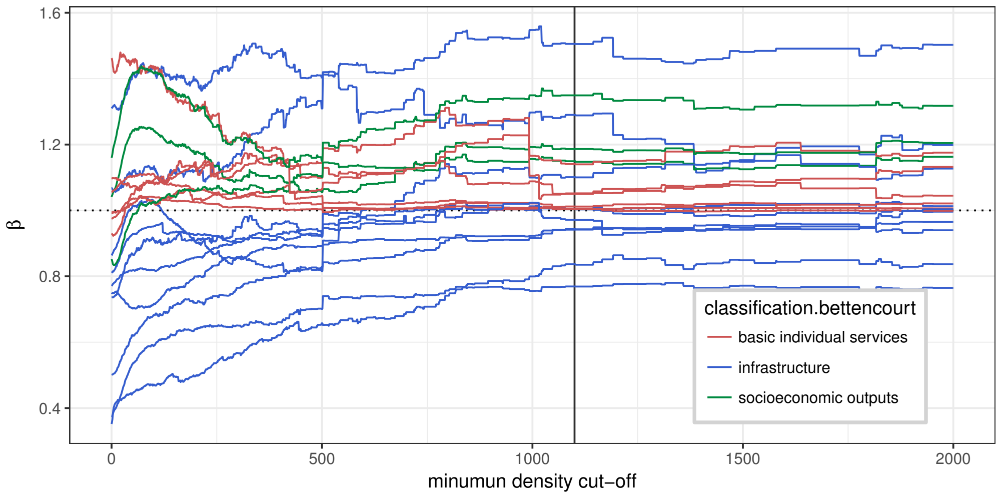

Supplement: S2 Table — Values for β, and its 95% confidence interval refers to the final density cut-off value. Statistically insignificant variables are not presented. (PDF) [file pone.0204574.s002.pdf]
