# Supplementary material for: Evolution of urban scaling: Evidence from Brazil
Source: PLoS One. 2018 Oct 4;13(10):e0204574. doi: 10.1371/journal.pone.0204574 (PMC6171854; doi:10.1371/journal.pone.0204574)

S2 Fig Exponents values for different urban indicators in the Brazilian urban system. Each dot represents the scaling exponent related to the best-fit line from the OLS regression of the population against the studied variable; vertical line segments represent 95% confidence interval (CI) of those regressions; colors are based on the proposed regime; the horizontal black-dotted line indicates linear relationship.

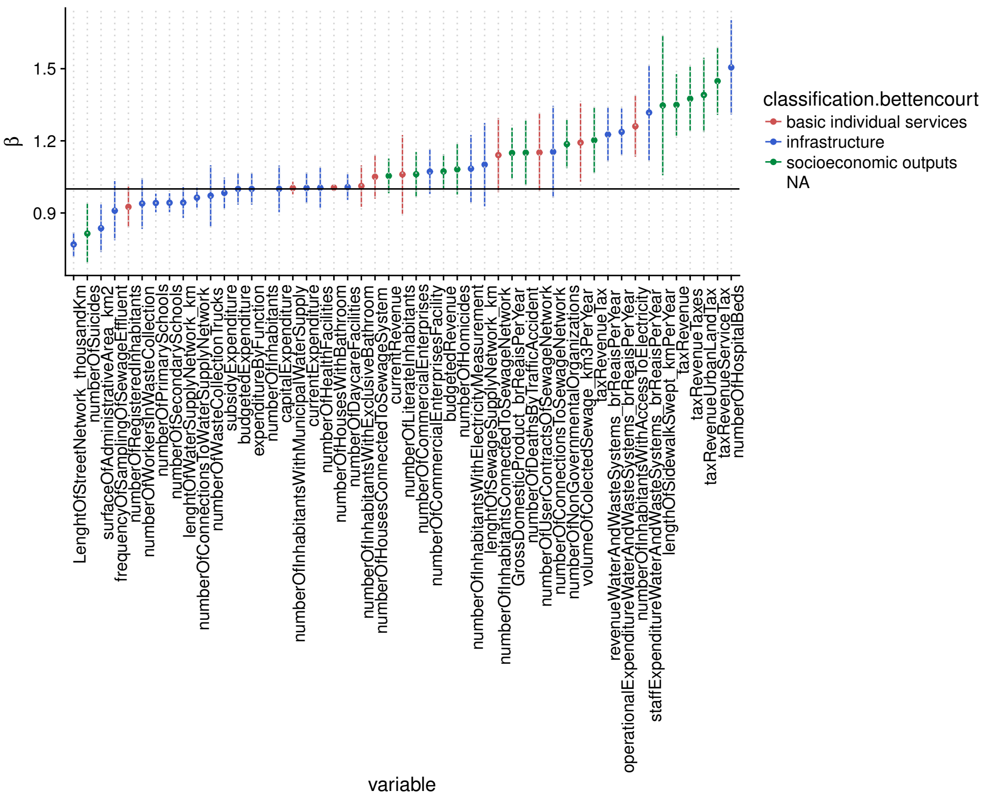

Supplement: S1 Fig — Each line represents the scaling exponent (y-axis) from OLS regressions of the log-transformed data of each variable as a function of the minimum density cut-off (x-axis). (PDF) [file pone.0204574.s003.pdf]
