# Supplementary material for: Evolution of urban scaling: Evidence from Brazil
Source: PLoS One. 2018 Oct 4;13(10):e0204574. doi: 10.1371/journal.pone.0204574 (PMC6171854; doi:10.1371/journal.pone.0204574)
Supplement: S2 Fig — Each dot represents the scaling exponent related to the best-fit line from the OLS regression of the population against the studied variable; vertical line segments represent 95% confidence interval (CI) of those regressions; colors are based on the proposed regime; the horizontal black-dotted line indicates linear relationship. (PDF) [file pone.0204574.s004.pdf]

S2 Table Deviations from the expected scaling regime proposed by Bettencourt [10] for all the statistically significant variables.. Values for β, and its 95% confidence interval refers to the final density cut-off value. Statistically insignificant variables are not presented.

Variable	β	Deviation
LenghtOfStreetNetwork	0.77 [0.72 , 0.82]	
numberOfSuicides	0.81 [0.69 , 0.94]	
surfaceOfAdministrativeArea	0.84 [0.74 , 0.94]	
frequencyOfSamplingOfSewageEffluent	0.91 [0.79 , 1.03]	
numberOfRegisteredInhabitants	0.93 [0.84 , 1.01]	X
numberOfWorkersInWasteCollection	0.94 [0.83 , 1.05]	
numberOfPrimarySchools	0.94 [0.90 , 0.98]	
numberOfSecondarySchools	0.94 [0.90 , 0.98]	
lenghtOfWaterSupplyNetwork_km	0.94 [0.88 , 1.01]	
numberOfConnectionsToWaterSupplyNetwork	0.96 [0.92 , 1.00]	
numberOfWasteCollectionTrucks	0.97 [0.84 , 1.10]	
subsidyExpenditure	0.98 [0.92 , 1.05]	X
budgetedExpenditure	1.00 [0.93 , 1.07]	X
expenditureByFunction	1.00 [0.93 , 1.07]	X
capitalExpenditure	1.00 [0.90 , 1.10]	X
numberOfInhabitantsWithMunicipalWaterSupply	1.00 [0.98 , 1.03]	
currentExpenditure	1.00 [0.94 , 1.07]	X
numberOfHealthFacilities	1.01 [0.92 , 1.09]	X
numberOfHousesWithBathroom	1.01 [0.99 , 1.02]	
numberOfDaycareFacilities	1.01 [0.95 , 1.06]	X
numberOfInhabitantsWithExclusiveBathroom	1.01 [0.93 , 1.10]	
numberOfHousesConnectedToSewageSystem	1.05 [0.96 , 1.14]	X
currentRevenue	1.05 [0.98 , 1.13]	
numberOfLiterateInhabitants	1.06 [0.90 , 1.22]	
numberOfCommercialEnterprises	1.06 [0.97 , 1.15]	
numberOfCommercialEnterprisesFacility	1.07 [0.98 , 1.16]	X
budgetedRevenue	1.07 [1,00 , 1.14]	
numberOfHomicides	1.08 [0.98 , 1.19]	
numberOfInhabitantsWithElectricityMeasurement	1.08 [0.94 , 1.22]	X
lenghtOfSewageSupplyNetwork	1.10 [0.93 , 1.27]	X
numberOfInhabitantsServedByWasteCollection	1.12 [1.04 , 1.19]	X
numberOfInhabitantsConnectedToSewageNetwork	1.14 [0.99 , 1.30]	X
GrossDomesticProduct	1.15 [1.04 , 1.25]	
numberOfDeathsByTrafficAccident	1.15 [1.02 , 1.28]	
numberOfUserContractsOfSewageNetwork	1.15 [0.99 , 1.31]	X
numberOfConnectionsToSewageNetwork	1.15 [0.96 , 1.35]	X
numberOfNonGovernmentalOrganizations	1.19 [1.09 , 1.29]	
volumeOfColectedSewage	1.19 [1.03 , 1.36]	X
taxRevenueTax	1.20 [1.07 , 1.34]	
revenueWaterAndWasteSystems	1.23 [1.11 , 1.34]	
operationalExpenditureWaterAndWasteSystems	1.24 [1.14 , 1.33]	X
numberOfInhabitantsWithAccessToElectricity	1.26 [1.13 , 1.39]	X
staffExpenditureWaterAndWasteSystems	1.32 [1.12 , 1.52]	X
lengthOfSidewalkSwept	1.35 [1.06 , 1.64]	
taxRevenue	1.35 [1.22 , 1.48]	
taxRevenueTaxes	1.38 [1.24 , 1.51]	
taxRevenueUrbanLandTax	1.39 [1.24 , 1.55]	
taxRevenueServiceTax	1.45 [1.31 , 1.59]	
numberOfHospitalBeds	1.51 [1.31 , 1.70]	X
